# Benefits of local consolidative treatment in oligometastases of solid cancers: a stepwise-hierarchical pooled analysis and systematic review

**DOI:** 10.1038/s41698-020-00141-4

**Published:** 2021-01-21

**Authors:** Chai Hong Rim, In-Soo Shin, Sunmin Park, Hye Yoon Lee

**Affiliations:** 1 Department of Radiation Oncology, Ansan Hospital, Korea University Medical College, Ansan, Gyeonggi-do Republic of Korea; 2 Graduate school of Education, Dongguk University, Seoul, Korea; 3 Department of General Surgery, Ansan Hospital, Korea University Medical College, Ansan, Gyeonggi-do Republic of Korea

**Keywords:** Metastasis, Surgical oncology, Radiotherapy

## Abstract

We conducted a meta-analysis of articles published in PubMed, MEDLINE, EMBASE, and Cochrane library to investigate the effectiveness of local consolidative therapy (LCT) against oligometastases. Data from randomized controlled trials (RCTs), balanced studies, and all studies combined were analyzed in a hierarchical manner. Pooled analyses of 31 studies (including seven randomized trials) investigating the effectiveness of LCT on overall survival revealed odds ratios of 3.04, 2.56, and 1.41 for all studies, balanced studies, and RCTs, respectively (all *p* < 0.05). The benefit of LCT was more prominent in patients with non-small cell lung and colorectal cancers than in those with prostate and small cell lung cancers. Moreover, the benefit of LCT was smaller in patients with high metastatic burdens (*p* = 0.054). In four of 12 studies with available information, additional grade ≥3 toxicities due to LCTs were reported. Overall, LCT is beneficial for patients with oligometastases, although such benefits are less evident in RCTs than in observational studies. Appropriate LCTs should be carefully selected considering their feasibility, disease type, and metastatic burden.

## Introduction

To date, cancer treatments have been selected depending on the pathologic stage of progression. The highest solid cancer stage indicates a systemic disease that has spread beyond the primary tumor and lymphatics and has little-to-no chance of being cured. Systemic administration of chemotherapy is regarded as the only valid option, while local modalities such as surgery or radiotherapy are deemed ineffective in terms of survival.

However, long-term survival is not uncommon among patients with metastases who have successfully undergone local salvage treatment. In the late twentieth century, a pivotal case series revealed that patients who underwent resection of the liver for metastases from colorectal cancer had a 5-year survival rate of 28–37%^1–3^; this rate reached 58% as reported in a more recent series^4^. The International Registry of Lung Metastases study revealed 5- and 10-year survival rates of 36% and 26%, respectively, after curative resection for lung metastases^5^. Survival outcomes were affected by smaller metastatic burdens or lower levels of tumor markers, which indicated the gradually progressing nature of the metastatic cascade and the presence of an intermediate state, that is, *oligometastasis*.

Nevertheless, more than two-thirds of such patients ultimately experience polymetastases, and open surgery might be burdensome for some patients whose chance of cure is uncertain and who are debilitated by their disease. The practical and clinical considerations of oligometastases have increased with technological advances in radiotherapy. Given the development of conformal technologies based on computed tomography planning, such as stereotactic body radiotherapy (SBRT), noninvasive, and ablative irradiation methods for metastatic lesions have become feasible^6^.

Extensive literature has recently emerged regarding the application of local consolidative treatment (LCT) for oligometastases^7,8^; however, the vast majority of publications are single-arm observational studies. This is partly because it can be difficult to design randomized controlled trials (RCTs) involving patients with metastases given ethical considerations (e.g., the possibility of missing a beneficial treatment because of assignment to a control arm) and patients’ widely varying clinical characteristics. The biological understanding of oligometastatic disease has evolved but remains unclear. Therefore, whether patients can benefit from local treatment for their metastases and whether oligometastasis exists as a status remains controversial^9,10^.

This meta-analysis aimed to assess the effectiveness of LCT for patients with oligometastases due to any type of solid cancer, thereby validating the benefit of LCT and aiding in clinical decision-making.

## Results

### Study selection and characteristics

**Fig. 1:**
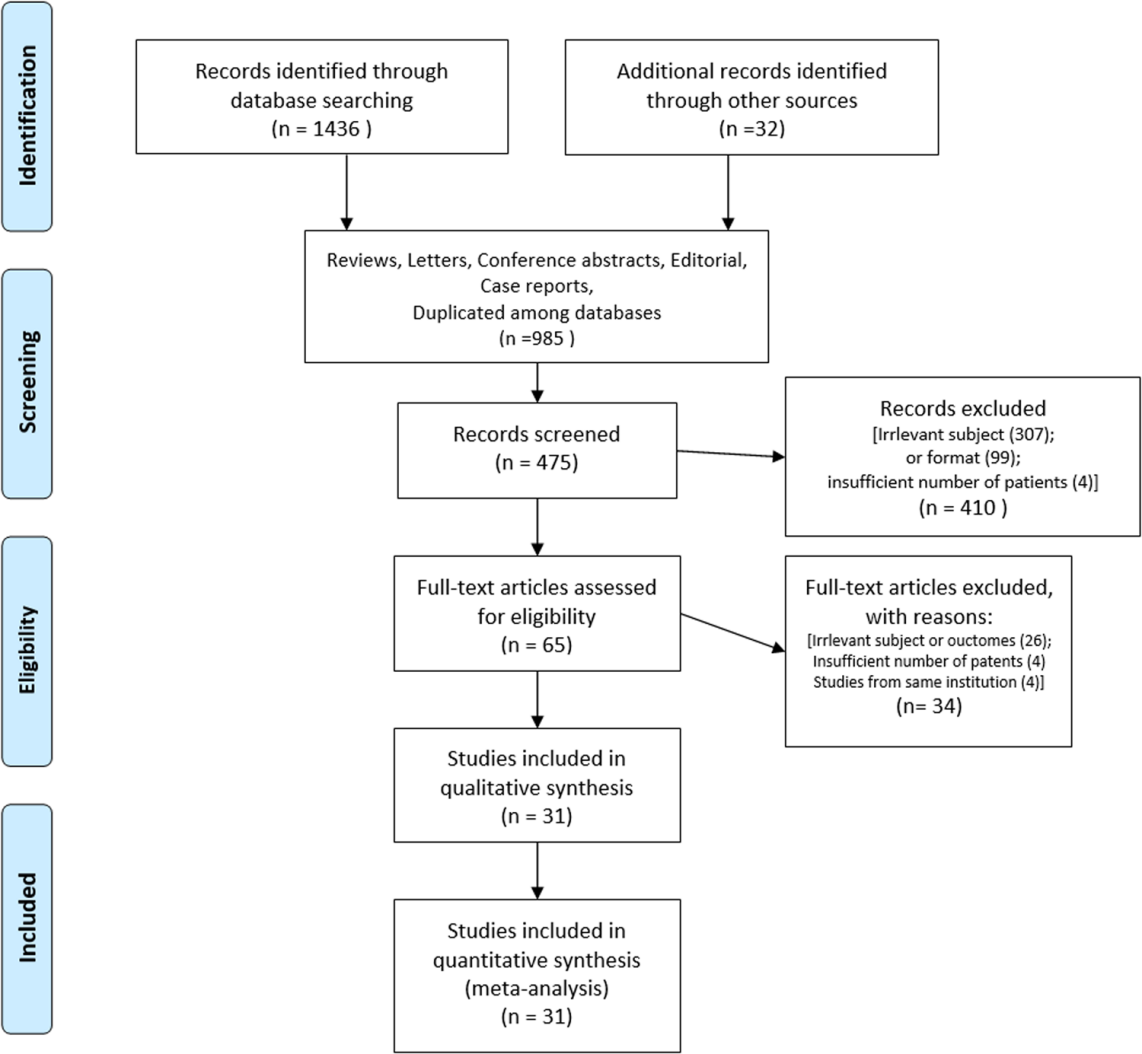
Study selection process: among the 1468 records intially searched, 31 studies were included in the current meta-analysis.

The meta-analysis included 31 controlled studies (23 retrospective and eight prospective studies)^9,11–40^ identified from 436 initially searched records in three databases; the studies included 4762 patients, of whom 2186 and 2576 were divided into the LCT and control arms, respectively. The study inclusion process is depicted in Fig. 1. Eight studies reported conflicts of interest with industrial sponsorship; the remainder had nothing to disclose. Seven studies were RCTs, eight used propensity score matching, 12 reported statistical comparisons of major clinical indicators between arms, and four had no comparative statistical data. Twelve studies included patients with non-small cell lung cancer (NSCLC), two included patients with small cell lung cancer (SCLC), six included patients with prostate cancer, three included patients with colorectal cancer, two included patients with esophageal cancer, two included patients with hepatocellular carcinoma (HCC), and one each included patients with the bile duct, head and neck, sarcoma, and multiple cancers. Most studies (25, 81%) included patients with synchronous and/or metachronous oligometastases and six (19%) targeted patients with metachronous oligometastases. Eleven studies (35%) defined oligometastases as the presence of ≤5 metastases; eight studies (26%) defined it as the presence of ≤3 metastases, and the remainder used varying definitions (Table 1; a detailed version is also provided in Supplementary Table 1).

**Table 1:** General information from the included studies.

First author, target disease	Patient recruitment years	Study type	LCT group compared with control	Total no. of patients	NOS score	Type of oligometastases; preceding Tx for primary dz.	Defined no. of oligomets.
He, NSCLC	2003–2013	R	N/A	21	7	Synchronous and metachronous; OP	≤3, in lung
Iyengar, NSCLC	2014–2016	P	RCT	29	9	Synchronous; PR or SD after CTx	Up to six lesions (including primary) in three organs
Sheu, NSCLC	1998–2012	R	PSM, balanced except higher age	74	9	Synchronous; no PD after CTx	≤3
Yano, NSCLC	1994–2004	R	N/A	93	7	Metachronous; surgery	Controllable with surgery or RTx
Frost, NSCLC	2000–2016	R	PSM	180	9	Synchronous	1–4 in one organ
Gomez, NSCLC	2012–2016	P	RCT	49	9	Synchronous and metachronous; CTx	≤3
Gray, NSCLC	2000–2011	R	Younger age (*p* = 0.027)	66	7	Synchronous	≤4, brain alone
Hu, NSCLC	2010–2016	R	More brain mets, less lung mets. (*p* < 0.001)	231	8	Synchronous; TKI	≤5 in single organ
Song, NSCLC	2005–2019	R	PSM, more peripheral location of mets. (*p* = 0.048)	70	9	Synchronous	≤5
Xu Q, NSCLC	2010–2016	R	Lower T and N stage	90	7	Synchronous; PR or SD after TKI	≤5
Ni, NSCLC	2015–2018	R	No significant difference	86	8	Synchronous	≤5
Shang, NSCLC (postop)	2005–2016	R	No significant difference except mets. location	152	8	Synchronous	≤5
Xu, SCLC (extended)	2010–2015	R	PSM, more weight loss patient	44	9	Synchronous	In one organ or in single RT portal
Bouman-Wammes, prostate	2009–2015	R	Higher PSA at Dx. (*p* = 0.015), more single mets (*p* = 0.003)	63	7	Metachronous; prostatectomy or RTx	≤3
Lan, prostate	2005–2016	R	Lower PSA (*p* = 0.003), cT (*p* < 0.001), N stage (*p* = 0.015), fewer bone mets (*p* = 0.019)	111	7	Synchronous	≤5
Ost, prostate	2012–2015	P	RCT	62	9	Metachronous; OP, RTx	≤3
Steuber, prostate	1993–2014	R	PSM	659	9	Metachronous; OP and adjuvant RTx (biochemical failure)	≤5
Parker, prostate	2013–2016	P	RCT	819	9	Synchronous	≤3 (low-burden subgroup)
Tsumura, prostate	2003–2013	R	N/A	40	7	Synchronous	≤5
Giessen, colorectal	2000–2004	P	More N-, better PS	253	7	Synchronous and metachronous; OP (95%)	1 (~95% of patients)
Ruers, colorectal	2002–2007	P	RCT	119	9	Synchronous and metachronous	≤9, all resectable or ablatable
Ruo, colorectal	1996–1999	R	More comorbidity (*p* = 0.04), more liver only and single mets. (*p* = 0.02)	230	7	Synchronous	≤3
Palma, multiple	2012–2016	P	RCT	99	9	Metachronous; no progression after definitive Tx	≤5
Chen Y, esophagus	2012–2015	R	No significant difference	461	8	Synchronous	≤3
Depypere, esophagus	2002–2015	R	N/A	20	7	Synchronous or metachronous; NAC(R)T	3–5 mets in single organ
Chen J, HCC	2013–2016	R	PSM	68	9	Synchronous	≤5 in lung
Pan, HCC	2004–2013	R	PSM	92	9	Synchronous	N/A
Morino, bile duct	1996–2015	R	PSM, more ICC (*p* < 0.001), more local mets. location (*p* = 0.005)	67	8	Metachronous; R0 or R1 resection	≤3
Schulz, head and neck	2001–2016	R	Intentioned match	47	7	Synchronous and metachronous; OP, CTx, RT	1 (77%), but ranged up to 10
Falk, sarcoma	2000–2012	R	Smaller primary tumor (*p* = 0.04), more controlled primary (*p* = 0.0003), less lung mets (*p* = 0.006)	281	7	Synchronous and metachronous; OP 93%, R0 62% R1 23%	≤5

*NOS* Newcastle-Ottawa Scale, *NSCLC* non-small cell lung cancer, *SCLC* small cell lung cancer, *HCC* hepatocellular carcinoma, *R* retrospective, N/A not assessable, *OP* operation, *P* prospective, *RCT* randomized controlled trial, *PR* partial remission, *SD* stable disease, *CTx* chemotherapy, *PSM* propensity score matching, *TKI* tyrosine kinase inhibitor, *PSA* prostate-specific antigen, *RTx* radiotherapy, *PS* performance status, *NACT* neoadjuvant chemotherapy, *NAC(R)T* neoadjuvant chemotherapy and/or radiotherapy.

LCT was performed principally to treat distant metastatic lesions as reported in 24 studies (77%) and to treat primary tumors in nine studies. Surgical resection was the LCT modality of choice in 19 studies (61%) and was performed exclusively in five studies and combined with other modalities in 14 studies (mostly radiotherapy in 12 studies). Radiotherapy was performed in 22 studies (71%), exclusively in nine studies and in combination with other modalities in 13 (mostly surgery, in 12 studies). Radiofrequency or microwave ablation was used in a few studies involving patients with liver neoplasms or metastases. Although only three studies reported significant differences in the number of metastases between the study arms, 12 of the 22 studies (55%) reported a higher frequency of single or low number metastases, without statistical significance, in the LCT arm. Clinical data from the studies are shown in Table 2 (with a more detailed version in Supplementary Table 2).

**Table 2:** Clinical information from the included studies.

First author, target disease	*N* (LCT arm)	No. of oligomets. (LCT arm)	Site (LCT arm)	Modality of LCT (LCT arm)	*N* (control arm)	No. of oligomets. (control arm)	Site (control arm)	Modality of control (control arm)	OS (LCT arm vs. control arm) 1/2-year rate	*P* value	PFS (LCT arm vs. control arm) 1/2-year rate	*p* Value
He, NSCLC	11	1 (60%); 2 (40%)	Lung 100%	Resection of mets. and/or CTx	10	N/A	Lung 100%	CTx	100/70% vs. 80/40%	0.026		
Iyengar, NSCLC	14	2 (50%); 3–4 (28.6%)	Lung or mediastinum >70%	SBRT and CTx	15	2 (40%); 3–4 (33%)	Lung or mediastinum >70%	CTx			1 year: 35.7 vs. 13.3%	0.01
Sheu, NSCLC	60	Mean 1.28	Brain (~50%)	Conventional RTx (76%)	14	Mean 1.23	Brain (~50%)	CTx	83.3/58.3% vs. 35.7/0%	<0.01	1 year: 46.7 vs. 18.2%	<0.01
Yano, NSCLC	44			Surgery or RTx and/or CTx	49			CTx or SOC	77.3/61.4% vs. 46.9/24.5%	<0.05		
Frost, NSCLC	90	1 (85%); 2 (8%)	Brain 57%; bone 10%; lung 9%	Lobectomy, CCRT, SBRT and 79% received CTx	90	1 (76%); 2 (14%)	Brain 32%; bone 22%; lung 21%	CTx (96%)	92.2/76% vs. 81.9/45.9%	<0.001	67.8/52.2% vs. 31/8.9%	<0.001
Gomez, NSCLC	25	0–1 (68%); 2–3 (32%)	Brain 28%; other 72%	RTx or surgery and standard maintenance	24	0–1 (62%); 2–3 (38%)	Brain 25%; other 75%	Standard maintenance	84/68% vs. 62.5/45.8%	0.017	52/28% vs. 20.8/12.5%	0.022
Gray, NSCLC	38	1 (50%); 2–4 (50%)	Brain 100%	Thoracic surgery or RTx, brain RTx, and CTx	28	1 (50%); 2–4 (50%)	Brain 100%	CTx and/or Brain RTx	71/54% vs. 46/26%	<0.001		
Hu, NSCLC	143	1–3 (81%); 4–5 (19%)	Brain 44%; bone 35%	Surgery and/or radiotherapy and TKI	88	1–3 (83%); 4–5 (17%)	Bone 42%; lung 33%	CTx (TKI)	95.3/72.1% vs. 84.1/40.9%	0.001	60.7/18.6% vs. 33.3/10.8%	<0.001
Song, NSCLC	35	1 (46%); 2 (29%); 3-5 (26%)	Lung 57%; bone 40%; liver 30%	Surgery or RTx and CTx	35	1 (23%); 2 (40%); 3–5 (37%)	Lung 60%; bone 54%	CTx	51.4/28.6% vs. 31.4/5.7%	0.002		
Xu Q, NSCLC	51	1 (49%); 2–3 (51%)		Surgery or RTx after TKI	39	1 (41%); 2–3 (51.3%)		CTx (TKI)		<0.001	86.3/25.6% vs. 70.5/0%	<0.001
Ni, NSCLC	34	1–3 (85%); 4–5(15%)	Lung 40%; liver 23%; adrenal gland 16%	TKI and MWA	52	1–3 (89%); 4–5 (11%)	Lung (32%); bone (23%); liver (20%)	CTx (TKI)	94.1/67.6% vs. 90.3/46.2%	0.04	88.2/23.5% vs. 61.5/0%	0.02
Shang, NSCLC (postop)	105	1 (73%); 2–5 (27%)	LN 46%; brain 24%; lung 19%	RTx or RFA and/or CTx	47	1 (72%); 2–5 (28%)	LN (72%) lung (32%)	CTx or BSC	1 year: 72.4 vs. 72.3%	0.519	1 year: 40.9 vs. 29.8%	0.006
Gore, SCLC (extended)	44	1 (32%); 2–4 (68%)	Adrenal 25%; distant LN 23%: liver 23%	PCI and cRTx	42	1 (41%); 2–4 (60%)	Distant LN 31%; Bone 26%; Liver 24%	PCI	1 year: 50.8 vs. 60.1%	0.21	1 year: 23.9 vs. 20.5%	0.01
Xu SCLC (extended)	22			RTx and CTx	22			CTx	72.7/25.2% vs. 18.2/12.7%	0.002	40.9/19.3% vs. 9.1/4.8%	0.006
Bouman-Wammes, prostate	43	1 (81%); 2 (14%)	LN 77%; bone 21%	SBRT	20	1 (45%); 2 (40%)	LN 65%; Bone 35%	Active surveillance			72.1/35.8% vs. 22.6/0%	<0.001
Lan, prostate	35	1 (26%) 2 (37%) 3 (20%)	Bone 100%	Prostatectomy and ADT	76	1 (8%) 2 (32%) 3 (30%)	Bone 100%	ADT	CSS 3/5 years: 90.8/63.6% vs. 87.9/74.9%	0.773	82.8/62.8% vs. 65.8/38.2%	0.184
Ost, prostate	31	1 (58%); 2 (19%); 3 (22%)	LN 55%; non-nodal 45%	SBRT (81%) or resection	31	1 (29%); 2 (32%); 3 (39%)	LN 55%; non-nodal 45%	Active surveillance			70.9/45.2% vs. 64.5/32.3%	0.11
Steuber, prostate	165		Pelvic LN ~90%	PLND or SBRT and ADT	494		Pelvic LN ~90%	ADT	OS 3/5 years: 99.2/98.7 vs. 98.2/95.4%	0.23		
Parker, prostate	410		Bone 76%; distant LN 36%	RT and ADT	409		Bone 76%; distant LN 34%	ADT	OS 1/2/3 years: 98.8/92.5/82.6 vs. 96.7/87.7/74.8%	0.007	89.6/72.8% vs. 86.3/69.6%	0.033
Tsumura, prostate	22		Bone or pelvic LN	Metastatic RTx, prostate brachy, and HTx	18		Bone or pelvic LN	Prostate brachy and HTx			94.4/88.9% vs. 95.5/73.3%	0.0269
Giessen, colorectal	38	1 (95%)	Liver 100%	Hepatic resection and CTx	215	1 (100%)	Liver 100%	CTx	97.4/89.5% vs. 68/37.6%	<0.001	63.2/36.8% vs. 21.2/5.2%	<0.001
Ruer, colorectal	60	1–3 (48%); 4–6 (30%); 7–9 (22%)	Liver 100%	RFA, surgery and/or CTx	59	1–3 (31%); 4–6 (46%); 7–9 (24%)	Liver 100%	CTx	91.7/75% vs. 89.8/74.5%	0.01	58.3/35% vs. 40.7/20.3%	0.005
Ruo, colorectal	127	1 (68%); 2 (26%); 3 (6%)	Liver 56%	Bowel surgery and CTx	103	1 (53%); 2 (30%); 3 (17%)	Liver 41%	CTx (83.5%)	63.8/25% vs. 35.9/6%	<0.001		
Palma, multiple	66	1 (46%); 2 (29%); 3(18%)	Lung 43%; bone 35%	SBRT and/or standard CTx	33	1 (36%); 2 (40%); 3 (18%)	Lung 53%; bone 31%	CTx	84.3/69.7% vs. 87.4/60.6%	0.09	54.5/36.4% vs. 22.7/15.2%	0.0012
Chen Y, esophagus	196			CCRT	265			CTx	72.8/27.2% vs. 63.5/17.5%	0.056	27.6/4.7% vs. 21.9/0.9%	0.002
Depypere, esophagus	10		Lung 50%; adrenal 20%	Esophagectomy ± lung metastatectomy	10		Liver 50%; brain 30%	CTx	80/40% vs. 50/10%	0.042		
Chen J, HCC	34		Lung 100%	TACE, RFA, resection, and sorafenib	34		Lung 100%	Sorafenib	67.6/47% vs. 35.3/23.5%	0.015	(TTP) 11.8/0% vs. 0/0%	0.009
Pan, HCC	46	Mean 2.22 ± 1.35	LN 100%	RFA and BSC or sorafenib	46	Mean 2.74 ± 1.37	LN 100%	BSC or sorafenib	58.3%/11.7% vs. 17.9/0%	0.001		
Morino, bile duct	33	Median 1 (1–-3)	Liver 39%; LN 27%; lung 12%	Surgery, RT, RFA, TACE, and/or CTx	34	Median 1 (1–3)	Local 35%; liver 29%; LN 21%	CTx or BSC	97/84.8% vs. 64.7/20.5%	<0.001		
Schulz, head and neck	37	1 (70%); 2–3 (16%)	Lung 59%; bone 22%	RTx or resection and/or CTx	10	1 (100%)	Lung 90%	CTx or BSC	67.6%/51.3% vs. 20%/10%	NA		
Falk, sarcoma	164		Lung 51%; liver 7%	RTx, RFA, OP ± CTx	117		Lung 69%; liver 7%	CTx in majority	79.6/63.6% vs. 52.3/36.3%	<0.0001		

*LCT* local consolidation therapy, *OS* overall survival, *PFS* progression-free survival, *CTx* chemotherapy, *M* metastases, *P* primary disease, *NSCLC* non-small cell lung cancer, *RTx* radiotherapy, *CCRT* concurrent chemoradiotherapy, *SBRT* stereotactic body radiotherapy, *ATT* aggressive thoracic therapy, *TKI* tyrosine kinase inhibitor, *MWA* microwave ablation, *SCLC* small cell lung cancer, *RFA* radiofrequency ablation, *LN* lymph node, *BSC* best supportive care, *PCI* prophylactic cranial irradiation, *ADT* androgen deprivation therapy, *PLND* pelvic lymph node dissection, *IMRT* intensity-modulated radiotherapy, *TACE* transarterial chemoradiotherapy, *TTP* time to progression, *OP* operation.

### Pooled analyses of primary endpoints

**Fig. 2:**
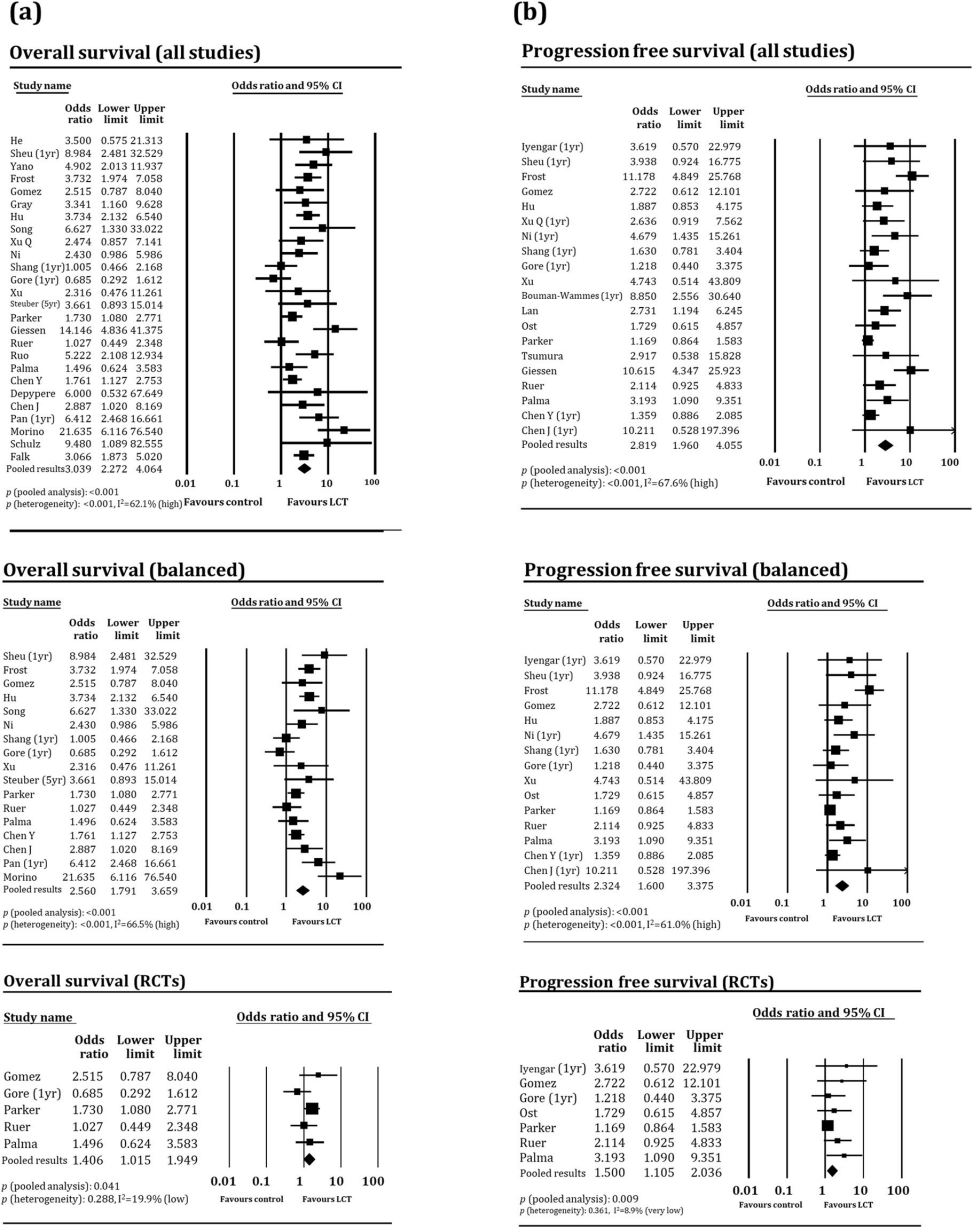
Forest plots of pooled analyses of primary endpoints. **a** Overall survival in all (top), balanced (middle), and randomized controlled trials (bottom) and **b** progression-free survival in all (top), balanced (middle), and randomized controlled trials (bottom). CI.

In the pooled analyses of overall survival (OS), the odds ratios (ORs) were 3.04 (95% confidence interval [CI]: 2.28–4.06, *p* < 0.001), 2.56 (95% CI: 1.79–3.66, *p* < 0.001), and 1.41 (95% CI: 1.02–1.95, *p* = 0.041) for all studies, balanced studies, and RCTs, respectively. In the pooled analyses of progression-free survival (PFS), the pooled ORs were 2.82 (95% CI: 1.96–4.06, *p* < 0.001), 2.32 (95% CI: 1.60–3.38, *p* < 0.001), and 1.39 (95% CI: 1.09–1.80, *p* = 0.009) for all studies, balanced studies, and RCTs, respectively. The pooled ORs for OS in studies principally targeting metastatic and primary tumors were 3.34 (95% CI: 2.40–4.66, *p* < 0.001) and 2.22 (95% CI: 1.21–4.08, *p* = 0.010), respectively, with no significant difference in subgroup comparisons (*p* = 0.248); the corresponding ORs for PFS were 3.34 (95% CI: 2.18–5.13) and 1.60 (95% CI: 0.99–2.59), respectively, with a significant difference between subgroups (*p* = 0.025). The pooled ORs for OS according to high versus low metastatic burden studies were 2.23 (95% CI: 1.56–3.20, *p* < 0.001) and 4.32 (95% CI: 2.45–7.59, *p* < 0.001), respectively, although the difference between these subgroups had a nonsignificant trend (*p* = 0.054). Regarding PFS, the ORs were 2.27 (95% CI: 1.67–3.09, *p* < 0.001) and 3.43 (95% CI: 1.70–6.96, *p* = 0.001), respectively, with no significant difference between the subgroups (*p* = 0.293). Heterogeneity was significant in most pooled analyses, but was low and insignificant in the pooled analyses of RCTs alone and in the pooled PFS analysis of the high metastatic burden subgroup. Possible publication biases were noted in the pooled analyses of OS in all studies and those investigating metastases, as well as in the pooled analyses of PFS in all studies, balanced studies, studies investigating metastases, and high metastatic burden studies. The main results are presented as Forest plots in Fig. 2, and the detailed results of pooled analysis are shown in Table 3.

**Table 3:** Pooled results of endpoints.

	No. of studies	No. of patients	Heterogeneity *p*	*I*^2^ (%)	Heterogeneity	Pooled results (OR, 95% CI)	*p* (pooled analyses)	Egger’s *p*	Trimmed value^a^
Overall survival									
All studies	26	2741	<0.001	62.1	High	3.04 (2.28–4.06)	<0.001	0.046	2.32 (1.71–3.15)
Balanced	17	2279	<0.001	66.5	High	2.56 (1.79–3.66)	<0.001	0.154	
RCTs	5	1172	0.288	19.9	Low	1.41 (1.02–1.95)	0.041		
Targeting metastases^b^	20	3146	<0.001	61.6	High	3.34 (2.40–4.66)	<0.001	0.080	2.41 (1.68–3.44)
Targeting primary disease^b^	6	1311	0.028	60.1	High	2.22 (1.21–4.08)	0.010		
High metastatic burden^c^	14	2074	0.017	49.9	Moderate	2.23 (1.56–3.20)	<0.001	0.674	
Low metastatic burden^c^	9	2154	<0.001	75.6	Very high	4.32 (2.45–7.59)	<0.001		
NSCLC	11	1112	0.168	29.1	Moderate	3.14 (2.24–4.41)	<0.001	0.613	
SCLC	2	130	0.184	43.2	Moderate	1.04 (0.34–3.24)	0.942		
Prostate	2	1478	0.323	~0	Very low	1.87 (1.19–2.92)	0.006		
Colorectal	3	602	<0.001	87.3	Very high	4.11 (0.91–18.5)	0.066		
Progression-free survival									
All studies	20	3116	<0.001	67.6	High	2.82 (1.96–4.06)	<0.001	0.001	1.59 (1.07–2.34)
Balanced	15	2559	0.001	61.0	High	2.32 (1.60–3.38)	<0.001	0.006	1.48 (0.99–2.22)
RCTs	7	1263	0.361	8.9	Very low	1.39 (1.09–1.80)	0.009		
Targeting metastases^b^	16	2010	0.001	62.0	High	3.34 (2.18–5.13)	<0.001	0.043	1.83 (1.14–2.96)
Targeting primary disease^b^	4	1106	0.155	42.8	Moderate	1.60 (0.99–2.59)	0.056		
High metastatic burden^c^	11	1111	0.827	~0.0	Very low	2.27 (1.67–3.09)	<0.001	0.04	1.99 (1.50–2.64)
Low metastatic burden^c^	9	1961	<0.001	86.2	Very high	3.43 (1.70–6.96)	0.001		
NSCLC	8	891	0.048	50.7	Moderate	3.28 (1.91–5.65)	<0.001		
SCLC	2	130	0.276	15.8	Low	1.65 (0.54–5.03)	0.376		
Prostate	5	1095	0.011	69.5	High	2.36 (1.15–4.82)	0.019		
Colorectal	2	372	0.009	85.2	Very high	4.69 (0.97–22.8)	0.055		

*OR* odds ratio, *CI* confidence interval, *RCT* randomized controlled trial, *NSCLC* non-small cell lung cancer, *SCLC* small cell lung cancer, *HCC* hepatocellular carcinoma.

Pooled analysis was not performed for diseases with only one eligible study.

^a^Values from Duval and Tweedie’s trim and fill method.

^b^Categorized according to the intended goal of local consolidation therapy and primarily targeted lesions

^c^Studies in which >80% of patients had a single metastasis or those that allowed patients with three or fewer metastases were regarded as low-burden studies; otherwise, studies that did not meet these criteria were regarded as high-burden studies (e.g., studies including patients with ≤5 metastases).

In the pooled analyses of OS according to cancer types, the benefit of LCT was more prominent in patients with NSCLC (OR: 3.14, *p* < 0.001; pooled 2-year OS: 65.2 vs. 37.0%) and colorectal cancer (OR: 4.11, *p* = 0.066; 2-year OS: 66.2 vs. 33.2%) than in those with prostate cancer (OR: 1.87, *p* = 0.006; 3-year OS: 95.6 vs. 92.6%) and SCLC (OR: 1.04, *p* = 0.942; 60.7 vs. 42.8%). Heterogeneity was not significant in the pooled analyses of OS for patients with NSCLC, SCLC, and prostate cancer but was significant in the pooled analyses of OS for those with colorectal cancer. Similar results were obtained for the pooled analyses of PFS; the benefit of LCT was higher for patients with NSCLC (OR: 3.28, *p* < 0.001; pooled 2-year PFS: 28.9 vs. 8.6%) and colorectal cancer (OR: 4.69, *p* = 0.055; 2-year PFS: 35.7 vs. 10.5%) and was lower for those with prostate cancer (OR: 2.36, *p* = 0.019, 2-year PFS: 82.7 vs. 61.7%) and SCLC (OR: 1.65, *p* = 0.376; 1-year PFS: 30.9 vs. 16.6%). Heterogeneity was not significant in the pooled analyses of PFS for patients with SCLC but was significant for those with NSCLC and those with prostate and colorectal cancers. Detailed results according to the disease type are shown in Tables 3 and 4.

**Table 4:** Pooled temporal analyses of numerical overall and progression-free survival.

Disease/ overall survival	No. of studies	No. of patients	Pooled results, LCT vs. control (95% confidence interval)
Overall survival			
NSCLC			
1-year OS	11	1112	85.0% (75.8–91.1) vs. 69.4 (54.4–81.1)
2-year OS	10	960	65.2% (55.5–73.7) vs. 37.0 (26.7–48.6)
Colorectal			
1-year OS	3	602	88.1% (57.0–97.7) vs. 67.5% (37.7–87.7)
2-year OS	3	602	66.2% (22.4–93.0) vs. 33.2% (8.8–71.9)
Prostate			
3-year OS	2	1477	95.6% (47.1–99.8) vs. 92.6% (41.9–99.5)
SCLC			
1-year OS	2	130	60.7% (38.1–79.4) vs. 42.8 (14.7–76.4)
Progression-free survival			
NSCLC			
1-year PFS	8	891	61.3% (48.7–72.6) vs. 35.7% (23.9–49.6)
2-year PFS	5	636	28.9% (16.8–45.0) vs. 8.6% (5–14.5)
Colorectal			
1-year PFS	2	372	60.2% (50.2–69.4) vs. 29.5% (14.2–51.4)
2-year PFS	2	372	35.7% (26.9–45.6) vs. 10.5% (2.5–34.7)
Prostate			
1-year PFS	5	1095	82.7% (70.6–90.5) vs. 71.3% (44.3–88.5)
2-year PFS	5	1095	61.7% (42.8–77.6) vs. 45.9% (24.7–68.6)
SCLC			
1-year PFS	2	130	30.9% (17.2–49.2) vs. 16.6% (8.0–31.3)

*LCT* local consolidative treatment, *NSCLC* non-small cell lung cancer, *OS* overall survival, *HCC* hepatocellular carcinoma, *SCLC* small cell lung cancer, *PFS* progression-free survival.

### Complications

Twelve of 31 studies (38.7%) involving 2176 patients contained the data of complications related to treatment modalities. Palma et al.^40^ reported three grade 5 cases (4.5%) possibly related to SBRT, whereas Gore et al.^35^ reported a significantly higher rate of grade 3 toxicity (24.8 vs. 9.5%) in the LCT arm (with one patient developing grade 5 toxicity). Ruo et al.^36^ reported a serious postoperative morbidity rate of 20.5%, with two patients developing grade 5 complications within 30 days of elective colorectal surgery. Ni et al.^41^ reported that 9.3% of patients needed chest tube insertion, while no serious toxicities were reported in the control arm. Otherwise, no significant additional toxicities due to LCTs were reported in eight studies in which LCT consisted mainly of radiotherapy (Table 5).

**Table 5:** Assessment of complications.

First author, target disease	Modality of LCT	*n*	Control	*n*	Grade ≥3 toxicity
Iyengar, NSCLC	SBRT and CTx	14	CTx	15	A total of 7 (50%) and 9 (60%) cases for LCT and control, respectively; no G5 toxicity
Gomez, NSCLC	RT or surgery and standard maintenance	25	Standard maintenance	24	Two cases with G3 esophagitis in LCT; 1 G3 fatigue and 1 G3 anemia in control
Ni, NSCLC	TKI and MWA	34	TKI	52	Four (9.3%) of the MWA group needed chest tube drainage; no grade ≥3 toxicity related to TKI
Shang, NSCLC (postop)	RT or RFA and/or CTx	105	CTx or BSC	47	Overall: 24.8 vs. 21.2% (m/c Cx.: myelosuppression) 1 case (0.9%) of grade 5 (infection) in LCT arm
Gore, SCLC	PCI and cRT (45 Gy/15 F)	44	PCI	42	Overall: 25% vs. 9.5%; 1 case of grade 5 pneumonitis in LCT arm
Bouman-Wammes, prostate	SBRT (mostly 30 Gy/3 F or 35 Gy/7 F)	43	Active surveillance	20	No SBRT-related toxicity
Ost, prostate	SBRT (81%) or resection	31	Active surveillance	31	No grade ≥2 toxicity in LCT arm
Parker, prostate	RT and ADT	410	ADT	409	No data in low metastatic burden subgroup; (4 vs. 1% for whole population)
Tsumura, prostate	RT to metastases, prostate brachytherapy and HTx	22	Prostate brachytherapy and HTx	18	No difference in grade ≥2 toxicity
Ruo, colorectal	Bowel surgery and CTx	127	CTx (83.5%)	103	30-day operative mortality: 2 cases (1.6%); perioperative morbidity (20.5%)
Palma, multiple	SBRT and/or standard CTx	66	CTx	33	Higher rate in LCT (10.6% vs. 3%); 3 grade 5 cases due to SBRT
Chen Y, esophagus	CCRT (IMRT, 50 Gy/25 F to primary; 45 Gy/15 F to metastases; cisplatin/paclitaxel)	196	CTx	265	No significant difference between arms

*LCT* local consolidation therapy, *NSCLC* non-small cell lung cancer, *SBRT* stereotactic body radiotherapy, *CTx* chemotherapy, *RT* radiotherapy, *TKI* tyrosine kinase inhibitor, *MWA* microwave ablation, *BSC* best supportive care, *PCI* prophylactic cranial irradiation, *SCLC* small cell lung cancer, *cRT* chest radiotherapy, *ADT* androgen deprivation therapy, *HTx* hormone therapy, *OP* operation, *CCRT* concurrent chemoradiation, *IMRT* intensity-modulated radiotherapy.

## Discussion

The concept of *oligometastases* has attracted significant interest as a potentially curative opportunity for patients whose diseases were deemed intractable. Molecular studies that aim to identify disease-specific biomarkers or gene profiles to identify oligometastases have shown promising results^42,43^; however, external or internal validation was lacking or unsuccessful^10^. Clinical data reported to date are heterogeneous, making it difficult for physicians to decide whether or not to administer LCTs. Currently, decisions regarding the application of LCTs are made depending on single-arm studies that demonstrated favorable survival outcomes in select patients. However, complications arising from LCTs, the possibility of missed occult metastases, and the distribution of medical resources are issues for consideration^6,9^.

In the present meta-analysis, LCT was beneficial in terms of OS; the pooled results from all studies (OR: 3.04, *p* < 0.001) and balanced studies (i.e., those without significant differences in major clinical indicators; OR: 2.56, *p* < 0.001) were significant, with a high degree of heterogeneity. Possible publication biases were noted, and the trimmed value after sensitivity analysis was lower than the original value (OR: 2.32). The OR was also significant in the pooled analysis of RCTs (OR: 1.41, *p* = 0.041), with a low degree of heterogeneity, but it was lower in magnitude than the ORs of total and balanced studies. The pooled results of PFS also showed trends similar to those of OS. The significant results obtained from the pooled analyses of RCTs with respect to both OS and PFS support the application of LCT in oligometastatic settings. However, the extent of this benefit might be smaller than that derived from observational study findings, which mostly showed favorable survival outcomes in select patients^10^. The significant heterogeneity and possible publication biases additionally indicate that selection biases might be present in the literature, despite making efforts to balance both arms using statistical tests. For example, patients in the LCT arm of 12 of 22 studies (55%) with available information tended to have fewer numbers of metastases, although the differences were not significant.

Most of the clinical literature on oligometastases is disease specific, and only a few studies have compared outcomes among different cancer types. According to subgroup analyses based on cancer types, the benefits of LCT and survival outcomes vary among disease entities. The survival benefits of LCTs were the most prominent for patients with NSCLC and colorectal cancer. Of note, the benefit of LCTs in terms of OS and PFS in patients with colorectal cancer showed borderline significance in the pooled analyses (*p* = 0.066 and 0.055, respectively). However, considering that all three colorectal cancer studies individually showed a significant benefit in terms of OS or PFS^29,33,36^ and given that the long-term results of Ruers et al.’s study^29^ (in which the 5-year OS rates were 43.1% and 30.3% and the 5-year PFS rates were 24.4% and 5.9% in the LCT and non-LCT arms, respectively) were not reflected in the analyses, the pooled results should not be interpreted as nonsignificant. Although the benefit of LCT was significant for patients with prostate cancer, its magnitude was relatively small. Survival outcomes of patients with oligometastatic prostate cancer were favorable regardless of the application of LCTs, suggesting that prostate cancer has a less aggressive tumor biology than other cancer types^44^. The benefit of LCT was not significant for patients with SCLC in terms of either OS or PFS (*p* = 0.942 and 0.376, respectively). This finding was consistent with the conventional notion that SCLC behaves more like a systemic disease and metastasizes early^45^.

Regarding complications, additional grade ≥3 toxicities with LCTs were reported in four of 12 studies with available information, including seven cases of grade 5 toxicities. Among five studies of patients with lung cancer, two reported grade 5 toxicities^30,35^ and two had higher rates of serious complications after LCT^41^. In the colorectal cancer study conducted by Ruo et al.^36^ bowel surgery resulted in additional complications, including two cases of 30-day mortality and serious perioperative morbidity (20.5%). In comparison, additional serious toxicities due to LCTs were rarely reported in prostate cancer studies^22,31,32,34^. Therefore, the application of LCTs for lung cancer, particularly in terms of technical planning and patient selection, should be performed with caution to minimize serious toxicities such as pneumonitis or esophagitis. Bowel surgery should be performed for patients whose clinical conditions allow it and in whom resection is feasible. Administering LCTs for oligometastatic prostate cancer was a relatively safe option. Because the adverse effects and oncologic benefits resulting from LCTs are different for each type of cancer, a tailored strategy for each patient is necessary considering the risk–benefit balance of LCT for oligometastatic diseases.

As observed in the included studies, the definition of oligometastasis varies. Some studies allowed for ≤3 metastases, some studies allowed for ≤5, and a few studies selected patients based on the ability of LCT to cover the metastases. Given clinical heterogeneities, it is difficult to set a clear cut-off number for metastases for determining the benefit of LCTs, even though clinical and biological differences are apparently present between oligometastatic and polymetastatic statuses^46,47^. Parker et al.^32^ reported that LCT was beneficial only for patients with low metastatic burdens (≤3 metastases) and not for those with higher metastatic burdens. In the same vein, our study revealed lower ORs in the high metastatic burden subgroup than in the low metastatic burden subgroup with a borderline significant difference (*p* = 0.054). Little is known about whether LCT that targets the primary disease is as beneficial as that which targets all the oligometastatic foci; other than for nephrectomy and metastatic renal cell carcinomas, data regarding LCT benefit are mostly preclinical or exploratory^48^. Although the OS benefit was not significantly different in subgroup comparisons, the PFS benefit differed among studies investigating primary diseases vs. those examining metastases. Our hypothesis regarding this PFS benefit is that LCTs covering metastatic lesions might have additional oncologic benefits over systemic treatment and that the studies that principally investigated primary tumors might have involved more patients with uncontrolled primary disease than did the other studies. The meta-analysis methodology is limited in its ability to evaluate the causes of the aforementioned differences. However, our results will aid in clinical decision-making in clinical practice and will lead to hypotheses for future oligometastasis research to identify differences among cancer types and define LCT targets.

We included studies with multiple cancer types, which is not an uncommon approach in investigations of LCTs for oligometastases^40^. This might cause heterogeneity to some extent among studies that affect the pooled analyses. However, this might also be a method to test the hypothesis that many cancers share an intermediate metastatic cascade called oligometastasis. In addition, this method overcomes the limitation of the small number of studies available for each specific cancer type. To improve the quality of our analyses and results, we rigorously evaluated and interpreted heterogeneity based on statistical methods and performed various subgroup analyses and stepwise analyses according to the studies’ quality. Other limitations include the small number of available studies involving patients with diseases other than NSCLC, prostate cancer, and colorectal cancer, as well as the methodological limitations of meta-analyses in that only outcomes, but not causes, can be determined.

In conclusion, our study demonstrated the oncologic benefits of LCTs in oligometastatic settings in terms of both OS and PFS. Although benefits were also observed when analyzing RCTs, their extent was smaller than that expected from literature data that included observational studies. LCT benefits were more prominent for oligometastases from NSCLC and colorectal cancer. Additional grade ≥3 complications due to LCT were found in approximately one-third of studies with available information. Patients with low metastatic burdens can derive greater benefits from LCTs. Therefore, appropriate LCTs should be selected carefully considering patients’ clinical conditions and disease types. Future research is warranted to identify the oligometastatic conditions in which LCTs are most likely to provide benefit and to investigate the underlying biology of oligometastases with respect to the benefits of LCT.

## Methods

### Study protocol

Our study adhered to the Preferred Reporting Items for Systematic Reviews and Meta-Analysis guidelines. The meta-analysis was designed to answer the following PICO question: “Does LCT confer an oncologic benefit for patients with oligometastases?” By implication, the response to this question would demonstrate whether a clinically meaningful “oligometastatic” status exists. LCT was defined as any local treatment targeted toward metastases and/or remnant primary disease in an oligometastatic setting. PubMed, MEDLINE, EMBASE, and Cochrane library were systematically searched by two independent reviewers for articles published up to March 4, 2020. The following search terms were used with no language restrictions: (oligometastasis OR oligometastases OR oligometastatic OR “limited metastatic” OR “limited metastasis” OR “limited metastases”) AND survival AND (randomized OR randomized OR versus OR comparison OR compare OR controlled). The reference lists of the extracted articles were also searched. Details of the searching strategy are shown in Supplementary Note 1. We compared the data of the LCT and control arms in the retrieved published studies; studies published before 2000 were excluded to avoid introducing potential bias from outdated treatments. Online registration of the protocol was not performed.

### Selection criteria

The inclusion criteria were as follows: (1) controlled trials (including both randomized and non-randomized) involving patients with oligometastases that compared the outcomes of those who underwent LCT with the outcomes of those in the control group, (2) ≥10 patients in each arm, (3) at least one primary endpoint provided, and (4) oligometastases defined as ≤5 metastases or as metastases that could definitely be encompassed and treated with LCT. The primary endpoints were OS and PFS. Grade ≥3 complications related to LCTs were assessed subjectively. For multiple studies published from a single institution, only those with a larger number of patients and no (or negligible) overlapping patient pools were included. Duplicate studies and those with irrelevant formats (e.g., reviews, editorials, letters, or case reports) were automatically filtered. Full-text reviews were performed to identify studies that fulfilled the inclusion criteria.

### Data extraction and quality assessment

Data were extracted using a pre-standardized form; PFS and OS data were estimated from descriptive graphs in the absence of numerical reports. Quality assessment was performed using the Newcastle-Ottawa Scale^49^ for cohort studies. Among the three scale domains (“selection” [four points], “comparability” [two points], and “outcome” [three points]), the difference in scores among the studies was mostly due to “comparability.” To avoid subjectivity, we defined the rationale for evaluating comparability based on discussion between clinical oncologists and a biostatistician on the following topics: (1) RCTs were assigned a full score (two points) unless they had serious clinical differences between the comparison arms or flaws in their study designs; (2) statistically matched cohorts (e.g., propensity score matching) or cohorts without significant differences in major clinical indicators were assigned one point; and (3) those with no statistical comparisons or no possibility of clinically significant differences between arms were allotted zero points. Major clinical indicators included the number of metastases, performance status, age, T stage, N stage, prostate-specific antigen (for prostate cancer), and primary disease control; the locations of the metastases were not considered. Studies that scored eight points or higher were considered to have high quality and balanced, while those with six or seven points were considered to have medium quality; lower scores were indicative of low quality.

### Statistical analyses

Pooled analyses of primary endpoints were performed (considering the study quality) in a stepwise-hierarchical manner. Overall analysis of all the studies was first performed; next, pooled analyses of balanced studies (eight points or higher on the Newcastle-Ottawa scale) were performed, followed by pooled analyses of the RCTs alone. Considering the varying study designs, treatment modalities, and clinical characteristics, the random-effects model was used for the first two analyses. While the fixed-effects model was used for the pooled analyses of RCTs. The 2-year OS and PFS rates were evaluated in pooled analysis: the 1-year rate was considered when the survival interval was too short or the 2-year rate neared 0% (e.g., patients with SCLC and HCC); the 3- or 5-year rates were considered if the survival rates were too high at 1 or 2 years (e.g., patients with prostate cancer). Pooled analyses of studies were also performed after categorizing them according to specific malignancies, LCT target (primary tumor vs. metastatic distant lesion), and metastatic burden using a random-effects model. Studies that enrolled >80% of patients with a single metastasis or those that included patients with ≤3 metastases were categorized as low-burden studies; otherwise, they were considered as high-burden (e.g., studies that enrolled patients with ≤5 metastasis were considered as high-burden studies). Heterogeneities were assessed using Cochran *Q*^50^ and *I*^2^ statistics^51^. Significant heterogeneity was considered to exist at *p* values <0.1 and *I*^2^ values ≥50%. The degree of heterogeneity was evaluated using the *I*^2^ values: 0–25% was considered indicative of low heterogeneity; 26–50%, moderate; 51–75%, high; and ≥76%, very high. *I*^2^ values <10% with *p* values <0.05 together indicated very low heterogeneity. Publication bias was evaluated using funnel plots and quantitatively using Egger’s test^52^. If a significant possibility of bias was detected (two-tailed *p* < 0.1)^52^, Duval and Tweedie’s trim and fill method^53^ was used for sensitivity analysis. Pooled temporal analyses of numerical OS and PFS rates according to the cancer type were performed using the *Q* test based on analysis of variance. Publication bias assessment was performed only for pooled analyses that included ≥10 studies. All statistical analyses were performed using Comprehensive Meta-Analysis software, version 3 (Biostat Inc., Englewood, NJ, USA).

### Ethical consideration

Ethical approval was not required because this study retrieved and synthesized using only previously published data.

### Reporting summary

Further information on research design is available in the Nature Research Reporting Summary linked to this article.

## Supplementary information

Supplementary materials

Reporting Summary Checklist

## Data Availability

This is a meta-analysis article that has used data retrieved directly from the text, figures, tables, and supplementary files of published articles. A list of all 31 articles used during this meta-analysis can be found in the following metadata record: 10.6084/m9.figshare.13292213^54^.

## Code availability

The authors declare that no custom code was used to generate the dataset. Statistical analyses were performed using Comprehensive Meta-Analysis software, version 3 (Biostat Inc., Englewood, NJ, USA).
