# Supplementary material for: Benefits of local consolidative treatment in oligometastases of solid cancers: a stepwise-hierarchical pooled analysis and systematic review
Source: NPJ Precis Oncol. 2021 Jan 21;5:2. doi: 10.1038/s41698-020-00141-4 (PMC7820397; doi:10.1038/s41698-020-00141-4)
Supplement: Supplementary file 1 — Supplementary materials [file 41698_2020_141_MOESM1_ESM.pdf]

Supplementary Note 1. Draft of search strategy

Search term: (oligometastasis OR oligometastases OR oligometastatic OR “limited metastatic” OR “limited metastasis” OR “limited metastases”) AND survival AND (randomised OR randomized OR versus OR comparison OR compare OR controlled)

Search strategy in Embase and Medline

Embase search:

#1 ('oligometastasis'/exp OR oligometastasis OR oligometastases OR oligometastatic OR 'limited metastatic' OR 'limited metastasis' OR 'limited metastases') AND ('survival'/exp OR survival) AND (randomised OR randomized OR versus OR 'comparison'/exp OR comparison OR compare OR controlled)

#2 ('oligometastasis'/exp OR oligometastasis OR oligometastases OR oligometastatic OR 'limited metastatic' OR 'limited metastasis' OR 'limited metastases') AND ('survival'/exp OR survival) AND (randomised OR randomized OR versus OR 'comparison'/exp OR comparison OR compare OR controlled) AND ('article'/it OR 'article in press'/it)

#3 ('oligometastasis'/exp OR oligometastasis OR oligometastases OR oligometastatic OR 'limited metastatic' OR 'limited metastasis' OR 'limited metastases') AND ('survival'/exp OR survival) AND (randomised OR randomized OR versus OR 'comparison'/exp OR comparison OR compare OR controlled) AND ('article'/it OR 'article in press'/it) NOT ('animal cell'/de OR 'animal model'/de OR 'animal tissue'/de OR 'case report'/de OR 'ex vivo study'/de OR 'human cell'/de OR 'human tissue'/de OR 'in vitro study'/de OR 'in vivo study'/de OR 'meta analysis'/de OR 'nonhuman'/de OR 'systematic review'/de)

*#1 is our basic strategy using Emtree; #2 is to filter studies with irrelevant formats (e.g. reviews, editorials, letters, conference abstracts); #3 is to filter studies which is not relevant clinical studies (e.g. case reports, in vivo studies, systematic reives)

Search strategy in Pubmed and Cochrane library

Using search query, filters to include clinical trial (I~IV), comparative study, clinical study, controlled clinical trial, multicenter study, and observational study were used. We did not use any filter in Cochrane library..

Supplementary table 1. General information from the included studies

Author, target disease	Affiliation	Publication Year	Patient recruit Year	Study type	LCT group compared to control	Total No. of patients	NOS score	Type of oligometastases; Preceding Tx. For primary dz.	Defined No. of oligomets.	Conflicts of interest
He, NSCLC	Sun Yat-sen University, China.	2017	2003–2013	R	N/A	21	7	Synchronous and metachronous; OP	≤3, in lung	None
Iyengar, NSCLC	University of Texas Southwestern, US	2017	2014–2016	P	RCT	29	9	Synchronous; PR or SD after CTx.	Up to 6 lesions (including primary) in 3 organs	None
Sheu, NSCLC	MDACC, US	2014	1998–2012	R	PSM, balanced except higher age	74	9	Synchronous; no PD after CTx.	≤3	None
Yano, NSCLC	Kyushu University, Japan	2010	1994–2004	R	N/A	93	7	Metachronous; surgery	Controllable with surgery or RTx	None
Frost, NSCLC	Charité, Evangelische Lungenklinik, DRK Klinikum Berlin-Mitte, Germany.	2018	2000-2016	R	PSM	180	9	Synchronous	1–4 in one organ	None
Gomez, NSCLC	MDACC, London health center, University of Colorado, US & UK	2019	2012–2016	P	RCT	49	9	Synchronous and metachronous; CTx.	≤3	None
Gray, NSCLC	Harvard Medical School, US	2014	2000–2011	R	younger age (p=0.027)	66	7	Synchronous	≤4, brain only	Industrial
Hu, NSCLC	Shanghai Jiaotong University, China.	2019	2010–2016	R	more brain mets, less lung mets. (P<0.001)	231	8	Synchronous; TKI	≤5 in single organ	None
Song, NSCLC	Cancer Hospital of China Medical University, Liaoning Cancer Hospital and Institute .	2020	2005–2019	R	PSM, more peripheral location of mets. (p=0.048)	70	9	Synchronous	≤5	None
Xu Q, NSCLC	Tongji University, China	2018	2010–2016	R	Lower T and N stage	90	7	Synchronous; PR or SD after TKI	≤5	None
Ni, NSCLC	Shandong First Medical University, China.	2020	2015–2018	R	no significant difference	86	8	Synchronous	≤5	None
Shang, NSCLC (postop)	Shandong University, China.	2019	2005–2016	R	no significant difference except mets. location	152	8	Synchronous	≤5	None
Gore, SCLC (extended)	57 centers	2017	2010–2015	P	RCT, more old age in control, p=0.03)	86	9	Synchronous; PR or CR after CTx.	≤4	Industrial
Xu SCLC (extended)	Tianjin Medical University, China	2017	2010–2015	R	PSM, more weight loss patient	44	9	Synchronous	in one organ or in single RT portal	None
Bouman-Wammes, prostate	VUMC, Netherland	2017	2009–2015	R	higher PSA at Dx. (p=0.015), more single mets (p=0.003)	63	7	Metachronous; prostatectomy or RTx.	≤3	Industrial
Lan, prostate	Lanzhou General Hospital of Lanzhou Command, China.	2019	2005–2016	R	lower PSA (p=0.003), cT (p <0.001), N stage (p=0.015), fewer bone mets (p=0.019)	111	7	Synchronous	≤5	None

Ost, prostate	Six institutions in Belgium	2018	2012–2015	P	RCT	62	9	Metachronous; OP, RTx.	≤3	Industrial
Steuber, prostate	Six European and one US center	2019	1993–2014	R	PSM	659	9	Metachronous; OP & adjuvant RTx (biochemical failure)	≤5	None
Parker, prostate	117 centers in UK and Swiss	2018	2013–2016	P	RCT	819	9	Synchronous	≤3 (low burden subgroup)	Industrial and government
Tsumura, prostate	Kitasato University, Japan.	2019	2003–2013	R	N/A	40	7	Synchronous	≤5	None
Giessen, colorectal	48 German centers	2013	2000–2004	P	more N-, better PS	253	7	Synchronous and metachronous; OP (95%)	1 (~95% of patients)	Industrial
Ruers, colorectal	22 European centers	2017	2002–2007	P	RCT	119	9	Synchronous and metachronous	≤9, all resectable or ablatable	None
Ruo, colorectal	Memorial Sloan Kettering Cancer Center, US	2003	1996–1999	R	more comorbidity (p=0.04), more liver only and single mets. (p=0.02)	230	7	Synchronous	≤3	None
Palma, multiple	10 institutions in Canada, Netherlands, Scotland, and Australia	2019	2012–2016	P	RCT	99	9	Metachronous; no progression after definitive Tx.	≤5	Industrial
Chen Y, esophagus	Wuhan, Zengzhou Univ, China	2019	2012–2015	R	no significant difference	461	8	Synchronous	≤3	None
Depypere, esophagus	University Hospitals Leuven, Belgium	2018	2002–2015	R	N/A	20	7	Synchronous or metachronous; NAC(R)T	3–5 mets in single organ	None
Chen J, HCC	Sun Yat-sen University Cancer Center, China.	2018	2013–2016	R	PSM	68	9	Synchronous	≤5 in lung	None
Pan, HCC	Sun Yat-sen University Cancer Center, China.	2017	2004–2013	R	PSM	92	9	Synchronous	N/A	None
Morino, bile duct	Kyoto University, Japan.	2020	1996–2015	R	PSM, more ICC (p<0·001), more local mets. location (p=0·005)	67	8	Metachronous; R0 or R1 resection	≤3	None
Schulz, head and neck	Klinikum rechts der Isar, Germany.	2018	2001–2016	R	intentioned match	47	7	Synchronous and metachronous; OP, CTx., RT	1 (77%), but ranged up to 10	None
Falk, sarcoma	15 centers, France	2015	2000–2012	R	smaller primary tumor (p=0·04), more controlled primary (p=0·0003), less lung mets (p=0·006)	281	7	Synchronous and metachronous; OP 93%, R0 62% R1 23%	≤5	Industrial

Abbreviations: NOS, Newcastle-Ottawa scale; NSCLC, non-small cell lung cancer; SCLC, small cell lung cancer; HCC, hepatocellular carcinoma; R, retrospective; N/A, not assessable; OP, operation; P, prospective; RCT, randomized controlled trial; PR, partial remission; SD, stable disease; CTx., chemotherapy; PSM, propensity score matching; TKI, tyrosine kinase inhibitor; PSA, prostate-specific antigen; RTx, radiotherapy; PS, performance status; NACT, neoadjvuant chemotherapy; NAC(R)T, neoadjuvant chemotherapy and/or radiotherapy

Supplementary table 2. Clinical information of included studies

Author, target disease	n	No. of oligomets.	Site	Target of LCT	Modality of LCT	n	No. of oligomets.	Site	Control	Median FU	OS (LCT arm vs. control arm)			PFS (LCT arm vs. control arm)		
	LCT arm					Control arm					Median (months)	1/2 year rate	*p*	Median (months)	1/2 year rate	*p*
He, NSCLC	11	1 (60%); 2 (40%)	Lung 100%	M	resection of mets. and/or CTx.	10	N/A	Lung 100%	CTx.	37.5	37 vs. 11.6	100/70% vs. 80/40%	0.026			
Iyengar, NSCLC	14	2 (50%); 3-4 (28.6%)	Lung or mediastinum >70%	M	SBRT & CTx.	15	2 (40%); 3-4(33%)	Lung or mediastinum >70%	CTx.	9.6	not reached			9.7 vs. 3.5	1yr: 35.7% vs 13.3%	0.01
Sheu, NSCLC	60	mean 1.28	Brain ~50%	M and P	conventiona RTx. (76%)	14	mean 1.23	Brain (~50%)	CTx.			83.3/58.3 vs. 35.7/0%	<0.01		1yr: 46.7% vs. 18.2%	<0.01
Yano, NSCLC	44			M (recurrence)	surgery or RTx. And/or CTx.	49			CTx. or SOC	~4 year	74 vs 10.9	77.3/61.4% vs. 46.9/24.5%	<0.05			
Frost, NSCLC	90	1 (85%); 2 (8%)	Brain 57%; bone 10%; lung 9%	M and/or P	Lobectomy, CCRT, SBRT;<br>79% received CTx.	90	1 (76%); 2 (14%)	Brain 32%; bone 22%; lung 21%	CTx. (96%)	32 vs. 19	60.4 vs 22.5	92.2/76 vs. 81.9/45.9%	<0.001	25.1 vs. 8.2	67.8/52.2% vs. 31/8.9%	<0.001
Gomez, NSCLC	25	0-1 (68%); 2-3 (32%)	Brain 28%; other 72%	M and/or P	RTx. or surgery & standard maintenance	24	0-1 (62%); 2-3 (38%)	Brain 25%; other 75%	Standard maintenance	38.8	41.2 vs. 17	84/68% vs. 62.5/45.8%	0.017	14.2 vs. 4.4	52/28% vs. 20.8/12.5%	0.022
Gray, NSCLC	38	1 (50%); 2-4 (50%)	Brain 100%	P	Thoracic surgery of RTx., brain RTx. & CTx	28	1 (50%); 2-4 (50%)	Brain 100%	CTx and/or Brain RTx.		26.4 vs. 10.5	71/54% vs. 46/26%	<0.001			
Hu, NSCLC	143	1-3 (81%); 4-5 (19%)	Brain 44%; Bone 35%	M	surgery and/or radiotherapy & TKI	88	1-3 (83%); 4-5 (17%)	Bone 42%; lung 33%	CTx. (TKI)	24	34 vs. 21	95.3/72.1% vs. 84.1/40.9%	0.001	15 vs 10	60.7/18.6% vs 33.3/10.8%	<0.001
Song, NSCLC	35	1 (46%); 2 (29%); 3-5 (26%)	Lung 57%; bone 40%; liver 30%	M and/or P	surgery or RTx. and CTx.	35	1 (23%); 2 (40%); 3-5 (37%)	Lung 60%; bone 54%	CTx.			51.4/28.6% vs. 31.4/5.7%	0.002			
Xu Q, NSCLC	51	1 (49%); 2-3 (51%)		P and/or M	surgery or RTx. After TKI.	39	1 (41%); 2-3 (51.3%)		CTx. (TKI)	38	40.9 vs. 30.8	96.1/86.3% vs. 94.9/71.8%	<0.001	20.6 vs. 13.9	86.3/25.6% vs. 70.5/0%	<0.001
Ni, NSCLC	34	1-3 (85%); 4-5(15%)	Lung 40%; liver 23%; adrenal gland 16%	M and/or P	TKI & MWA	52	1-3 (89%); 4-5 (11%)	Lung (32%); bone	CTx. (TKI)	36	34.8 vs. 22.7	94.1/67.6% vs. 90.3/46.2%	0.04	16.7 vs. 12.9	88.2/23.5% vs. 61.5/0%	0.02

								(23%); liver (20%)								
Shang, NSCLC (postop)	105	1 (73%); 2-5 (27%)	LN 46%; brain 24%; lung 19%	M and/or P	RTx. or RFA and/or CTx.	47	1 (72%); 2-5 (28%)	LN (72%) lung (32%)	CTx. or BSC	19	19 vs. 20	1yr: 72.4 vs 72.3%	0.519	10 vs. 7	1yr: 40.9 vs. 29.8%	0.006
Gore, SCLC (extended)	44	1 (32%); 2-4 (68%)	Adrenal 25%; distant LN 23%: liver 23%	P	PCI and cRTx.	42	1 (41%); 2-4 (60%)	Distant LN 31%; Bone 26%; Liver 24%	PCI	9	13.8 vs. 15.8	1yr: 50.8 vs. 60.1%	0.21	4.9 vs. 2.9	1yr: 23.9 vs. 20.5%	0.01
Xu SCLC (extended)	22			M and/or P	CTx and RTx	22			CTx.	36.4		72.7/25.2 vs. 18.2/12.7%	0.002		40.9/19.3 vs. 9.1/4.8%	0.006
Bouman-Wammes, prostate	43	1 (81%); 2 (14%)	LN 77%; bone 21%	M	SBRT	20	1 (45%); 2 (40%)	LN 65%; Bone 35%	Active surveillance					17.3 vs 4.2	72.1/35.8% vs. 22.6/0%	<0.001
Lan, prostate	35	1 (26%) 2 (37%) 3 (20%)	Bone 100%	P	Prostatectomy & ADT	76	1(8%) 2(32%) 3(30%)	Bone 100%	ADT	35		CSS 3/5yr: 90.8/63.6% vs. 87.9/74.9%	0.773	(PSA-RFS) 32 vs. 17	82.8/62.8% vs. 65.8/38.2%	0.184
Ost, prostate	31	1 (58%); 2(19%); 3(22%)	LN 55%; non-nodal 45%	M	SBRT(81%) or resection	31	1 (29%); 2 (32%); 3(39%)	LN 55%; non-nodal 45%	Active surveillance	3 year				(ADT-free survival) 21 vs. 13	70.9/45.2% vs. 64.5/32.3%	0.11
Steuber, prostate	165		Pelvic LN ~90%	M	PLND or and ADT	494		Pelvic LN ~90%	ADT			CSS 5/10 yr: 98.6/95.6 vs. 95.7/84.8% OS 3/5 yr: 99.2/98.7 vs. 98.2/95.4%	0.03; 0.23			
Parker, prostate	410		Bone 76%; distant LN 36%	P	RT and ADT	409		Bone 76%; distant LN 34%	ADT	37		1/2/3 yr: 98.8/92.5/82.6% vs. 96.7/87.7/74.8%	0.007		89.6/72.8% vs. 86.3/69.3%	0.033
Tsumura, prostate	22		Bone or pelvic LN	M	metastastic RTx., prostate brachy & HTx.	18		Bone or pelvic LN	prostate brachy& HTx.	62.5					94.4/88.9% vs. 95.5/73.3%	0.027
Giessen, colorectal	38	1 (95%)	Liver 100%	M	Hepatic resection and CTx.	215	1 (100%)	Liver 100%	CTx.		48.0 vs. 17.0	97.4/89.5% vs. 68/37.6%	<0.001	16.6 vs. 6.5	63.2/36.8% vs. 21.2/5.2%	<0.001
Ruer, colorectal	60	1-3 (48%); 4-6 (30%); 7-9 (22%)	Liver 100%	M	RFA, surgery and/or CTx.	59	1-3 (31%); 4-6 (46%); 7-9 (24%)	Liver 100%	CTx.	9.7 years	45.6 vs 40.5	91.7/75% vs. 89.8/74.5%	0.01	16.8 vs. 9.9	58.3/35% vs. 40.7/20.3%	0.005

Ruo, colorectal	127	1 (68%); 2(26%); 3(6%)	Liver 56%	P	bowel surgery and CTx.	103	1 (53%); 2(30%); 3(17%)	Liver 41%	CTx. (83.5%)		16 vs. 9	63.8/25% vs. 35.9/6%	<0.001			
Palma, multiple	66	1(46%); 2(29%); 3(18%)	lung 43%; bone 35%	M	SBRT and/or standard CTx.	33	1 (36%); 2(40%); 3(18%)	Lung 53%; bone 31%	CTx.	26 vs. 25	41 vs. 28	84.3/69.7% vs. 87.4/60.6%	0.09	12 vs. 6	54.5/36.4% vs. 22.7/15.2%	0.001
Chen Y, esophagus	196			M and P	CCRT	265			CTx	11.5	16.8 vs 14.8	72.8/27.2% vs. 63.5/17.5%	0.056	8.7 vs. 7.3	27.6/4.7% vs. 21.9/0.9%	0.002
Depypere, esophagus	10		Lung 50%; adrenal 20%	P	esophagectomy +/- lung metastatectomy	10		Liver 50%; brain 30%	CTx.		21.4 vs 12.1	80/40% vs. 50/10%	0.042			
Chen J, HCC	34		Lung 100%	M and/or P	TACE, RFA, resection & sorafenib	34		Lung 100%	Sorafenib	8.4	18.4 vs. 7.4	67.6/47% vs. 35.3/23.5%	0.015	TTP: 3.1 vs. 2.3	(TTP) 11.8/0% vs. 0/0%	0.009
Pan, HCC	46	Mean 2.22 +/- 1.35	LN 100%	M (lymph node)	RFA; and BSC or sorafenib	46	Mean 2.74 +/- 1.37	LN 100%	BSC or sorafenib	14 vs 13.8	13 vs. 7.8	58.3%/11.7% vs. 17.9/0%	0.001			
Morino, bile duct	33	Median 1 (1-3)	Liver 39%; LN 27%; lung 12%	M (recurrence)	Surgery, RT, RFA, TACE and/or CTx.	34	Median 1 (1-3)	Local 35.3%; liver 29%; LN 20.5%	CTx. or BSC	12.6	48.6 vs. 14.2	97/84.8% vs. 64.7/20.5%	<0.001			
Schulz, head and neck	37	1 (70%); 2-3 (16%)	Lung 59%; bone 22%	M	RTx. or resection and/or CTx.	10	1 (100%)	Lung 90%	CTx. or BSC		24 vs. 7	67.6%/51.3% vs. 20%/10%	NA			
Falk, sarcoma	164		Lung 51%; liver 7%	M	RTx., RFA, OP +/- CTx.	117		Lung 69%; liver 7%	CTx. in majority	25.7		79.6/63.6% vs. 52.3/36.3%	<0.0001			

Abbreviations: LCT, local consolidation therapy; OS, overall survival; PFS, progression free survival; CTx., chemotherapy; M, metastases; P, primary disease; NSCLC, non-small cell lung cancer; RTx., radiotherapy; CCRT, concurrent chemoradiothearpy; SBRT, stereotactic body radiotherapy; ATT, aggressive thoracic therapy; TKI, tyrosine kinase inhibitor; MWA, microwave ablation; SCLC, small cell lung cancer; RFA, radiofrequency ablation; LN, lymph node; BSC, best supportive care; PCI, prophylactic cranial irradiation; ADT, androgen deprivation therapy; PLND, pelvic lymph node dissection; IMRT, intensity modulated radiotherapy; TACE, transarterial chemoradiotherapy; TTP, time to progression; OP, operation
